# Establishment and evaluation of a nomogram predicting risks of missed diagnoses of colorectal polyps

**DOI:** 10.1186/s12876-022-02415-6

**Published:** 2022-07-11

**Authors:** Xiaobo Wang, Haiyang Guo, Yong Tang, Lin Chen, Xianfei Wang

**Affiliations:** Department of Gastroenterology, Shunqing District, Affiliated Hospital of North Sichuan Medical College, No.1 South Maoyuan Road, Nanchong City, 637000 Sichuan China

**Keywords:** Colorectal polyps, Missed diagnosis of polyps, Risk prediction model, Nomogram, Colorectal cancer

## Abstract

**Background:**

A missed diagnosis of colorectal polyps during colonoscopy may be associated with the occurrence of interval colorectal cancer. The risk factors for a missed diagnosis or a method to predict the risk of a missed diagnosis of colorectal polyps during colonoscopy remain unidentified.

**Methods:**

The clinical data of patients who underwent two colonoscopies within three months at the Affiliated Hospital of North Sichuan Medical College between February 2017 and August 2019 were retrospectively reviewed. Independent risk factors for missed diagnoses were identified, and a nomogram was established to predict the risk of missed diagnoses. The prediction performance of the nomogram was evaluated using C-index and calibration curves, and its clinical application value was assessed using the Youden index and decision curve analysis.

**Results:**

Independent influencing factors for missed diagnoses included age, endoscopist experience, bowel preparation, retroflected view, withdrawal time, number of polyps in the right colon, and number of polyps ≥ 6 mm. The C-index of the nomogram in the training and validation cohorts was 0.763 (95% confidence interval [*CI*]: 0.724 − 0.807) and 0.726 (95%*CI*: 0.657 − 0.794), respectively. The optimal cut-off value of the nomogram calculated using the Youden index was 152.2 points. Under the cut-off value, the sensitivity, specificity, positive predictive value, and negative predictive value were 67.1%, 75.7%, 45.8%, and 88.2%, respectively, in the training cohort, and 57.1%, 79.9%, 53.3%, and 82.3%, respectively, in the validation cohort.

**Conclusions:**

The nomogram provides a reference value for clinicians to analyse the risk of a missed diagnosis of colorectal polyps in individuals, identify high-risk groups, and formulate appropriate follow-up strategies.

## Background

Colorectal cancer (CRC) is the third most common type of cancer [1]. Colorectal polyps are recognized as precancerous lesions in colorectal cancer, and approximately 75 − 80% of colorectal cancer develops from colorectal polyps [2]. Colonoscopy is the preferred method for the detection and removal of colorectal polyps. However, missed diagnoses of colorectal polyps may occur to a varying degree during colonoscopy. For example, in a recent retrospective study [3], it was found that missed colorectal polyps on colonoscopy occurred 32.8% of the time, adenomas 25.6%, and advanced adenomas 10.4%. Some studies [4] have shown that every 1% increase in the detection rate of adenomas reduces the risk of interval colorectal cancer (CRC occurring after a negative colonoscopy) by 3% and the risk of fatal interval colorectal cancer by 5%. Therefore, identifying missed diagnoses of polyps following colonoscopy is significant in reducing the incidence of interval colorectal cancer.

Studies have confirmed that tandem colonoscopies or two or more consecutive colonoscopies within a short period are undoubtedly the best methods for detecting missed polyps [5]. However, considering the economic status and medical resources, it is impractical for all screened patients to undergo two or more colonoscopies. Low patient compliance is another important reason this measure cannot be clinically implemented. Therefore, it is necessary to evaluate the risk of missed diagnoses of colorectal polyps in an individual patient after a single colonoscopy based on relevant risk factors influencing missed polyps. Several studies [3, 6–8] have analysed the factors associated with missed diagnoses of colorectal polyps; however, only a few factors have been incorporated in these studies, and there are no predictive models able to predict the risk of missed colorectal polyps in individual patients.

This study retrospectively analysed the available clinical data to find the risk factors for missed diagnoses of colorectal polyps and establish and verify a nomogram to predict the risk of missed diagnoses of colorectal polyps.

## Materials and methods

### Patients

The clinical case and endoscopic data of patients who underwent two colonoscopies within three months at the Affiliated Hospital of North Sichuan Medical College between February 2017 and August 2019 were retrospectively reviewed. The first colonoscopy was performed to detect polyps, and the second was to remove them. Newly found polyps in the second colonoscopy were defined as missed polyps. Patients with missed polyps were categorized as the missed diagnosis group. Patients who underwent two colonoscopies between February 2017 and August 2018 were included in the training cohort to establish the nomogram. Patients who underwent a second colonoscopy or completed two colonoscopies between September 2018 and August 2019 were included in the validation cohort to validate the nomogram.

Inclusion criteria comprised the following: patients with a complete clinical case and endoscopic data; patients whose terminal ileum or ileocecal area was accessible in both colonoscopies; patients with good bowel preparation in the second colonoscopy and whose second colonoscopy was performed by an endoscopist who had performed ≥ 1000 colonoscopies; and patients with an interval of < 90 days between two colonoscopies. Exclusion criteria comprised the following: patients in whom the total number of polyps detected in the first colonoscopy was ≥ 15; patients with colorectal cancer; patients with inflammatory bowel disease; patients who had previously undergone bowel surgery; patients whose polyps could not be clearly described in terms of shape, location, quantity, and size; and patients who lacked clinical case or endoscopic data. The study was approved by the ethics committee, and informed consent was waived in view of the study's retrospective nature.

### Clinical data

The research variables in this study are reported in Table 1. Patient-related factors included age, sex, symptoms, diverticulum history, and family history of colorectal cancer (specifically, history of colorectal cancer in first-degree relatives). Factors related to endoscopic operation included the endoscopist’s experience, bowel preparation, sedation colonoscopy, retroflected view, and withdrawal time. Clinical characteristics of the polyps included the number of polyps in the first colonoscopy, number of polyps in the right colon, number of polyps in the left colon, number of rectal polyps, number of flat polyps, number of protruding polyps, number of polyps < 6 mm, and number of polyps ≥ 6 mm. The Boston Bowel Preparation Scale [9] was used to evaluate bowel preparation. According to the image recording time, the actual withdrawal time of the colonoscope was the time for the colonoscope to be withdrawn from the caecum to the anus minus the time for tissue biopsy. According to the Paris Classification [10], polyps are morphologically divided into two categories: protruding and flat. Polyp size was measured using the opening diameter of the biopsy forceps (6 mm). The cut-off value for the number of polyps was set as two since ≥ 2 polyps are commonly referred to as multiple polyps [11].

### Statistical methods

Statistical analyses were performed using SPSS v.26 and R4.0.1 software. Measurement data (continuous variables) were tested for normality using the Kolmogorov–Smirnov test. Measurement data that followed a normal distribution were expressed as($\overline{x} \pm s$)and analysed using an independent t-test. Measurement data that did not follow a normal distribution were expressed as the median (1/4–3/4 quantile) and analysed using the Mann − Whitney U test. Count data (categorical variables) were expressed as frequencies and percentages (n%) and analysed using the chi-square (χ^2^) test or Fisher's exact test. The potential influencing factors for missed diagnoses of colorectal polyps were screened using univariate logistic regression analysis in the training set. The multivariate logistic regression analysis included these potential influencing factors for evaluation. Simultaneously, Lasso regression was used to screen the influencing factors in the training set as double validation of the logistic regression analysis. The optimal parameter λ (corresponding to one standard error away from the minimum mean square error) was determined by tenfold cross-validation. Finally, the independent risk factors were introduced into R software (version R4.0.1). The RMS package was used to construct a nomogram for predicting the risk of missed diagnoses of colorectal polyps. The bootstrap method was used to repeat sampling 1,000 times to conduct internal validation of the nomogram model, and the validation dataset was used to conduct external validation of the nomogram model. The C-index and area under the receiver operating characteristic curve (AUC) were used to evaluate the discrimination of the nomogram. A calibration curve was used to evaluate the calibration performance. With the sensitivity, specificity, predictive values, and likelihood ratio under the optimal cut-off value (determined by the Youden index), a clinical decision curve was used to analyse the net income under different threshold probabilities to evaluate the clinical application value of the nomogram. The test levels α = 0.05 and *P* < 0.05 were considered statistically significant in all analyses.

## Results

### Clinical baseline characteristics of patients

A total of 992 patients were finally included in this study; among these, 699 patients were included in the training cohort, and 293 patients were included in the validation cohort according to the time of completion of the two colonoscopies. The baseline clinical characteristics of the patients in the training and validation cohorts are shown in Table 1. The baseline clinical characteristics of the two sets were similar. Only the number of polyps at the first colonoscopy, number of polyps ≥ 6 mm, and number of protruded polyps were significantly different between the groups (*P* < 0.05). After the first colonoscopy, the number of patients with missed polyps was 164 (23.5%) in the training cohort and 84 (28.7%) in the validation cohort.

**Table 1:** Baseline clinical characteristics of patients in the training and validation cohorts

Variable	Training cohort (n = 699)	Validation cohort (n = 293)	*P*
Age (years),$\overline{x} \pm SD$	53.7 ± 12.4	52.3 ± 12.3	0.113
Sex, n (%)			0.254
Male	441(63.1)	196(66.9)	
Female	258(36.9)	97(33.1)	
Symptom n (%)			0.126
No	202(28.9)	99(33.8)	
Yes	497(71.1)	194(66.2)	
Family history of CRC, n (%)			0.823
No	647(92.6)	270(92.2)	
Yes	52(7.4)	23(7.8)	
Diverticulum history, n (%)			0.885
No	646(92.4)	270(92.2)	
Yes	53(7.6)	23(7.8)	
Sedative colonoscopy, n (%)			0.317
No	427(61.1)	169(57.7)	
Yes	272(38.9)	124(42.3)	
Bowel preparation, n (%)			0.786
Adequate	565(80.8)	239(81.6)	
Poor	134(19.2)	54(18.4)	
Endoscopist’s experience, n (%)			0.417
≥ 1000	457(65.4)	181(61.8)	
500 − 1000	170(24.3)	83(28.3)	
< 500	72(10.3)	29(9.9)	
Retroflected view, n (%)			0.139
No	628(89.8)	272(92.8)	
Yes	71(10.2)	21(7.2)	
Withdrawal time, n (%)			0.449
≥ 6	421(60.2)	184(62.8)	
< 6	278(39.8)	109(37.2)	
No. of polyps at first colonoscopy, n (%)			0.031
< 2	327(46.8)	159(54.3)	
≥ 2	372(53.2)	134(45.7)	
No. of right colon polyps, n (%)			0.684
< 2	594(85.0)	246(84.0)	
≥ 2	105(15.0)	47(16.0)	
No. of left colon polyps, n (%)			0.126
< 2	576(82.4)	253(86.3)	
≥ 2	123(17.6)	40(13.7)	
No. of rectal polyps, n (%)			0.781
< 2	650(93.0)	271(92.5)	
≥ 2	49(7.0)	22(7.5)	
No. of polyps < 6 mm, n (%)			0.162
< 2	613(87.7)	266(90.8)	
≥ 2	86(12.3)	27(9.2)	
No. of polyps ≥ 6 mm, n (%)			0.017
< 2	508(72.7)	234(79.9)	
≥ 2	191(27.3)	59(20.1)	
No. of flat polyps, n (%)			0.409
< 2	619(88.6)	254(86.7)	
≥ 2	80(11.4)	39(13.3)	
No. of protruding polyps, n (%)			0.001
< 2	439(62.8)	217(74.1)	
≥ 2	260(37.2)	76(25.9)	
Interval,	21.6 ± 24.3	20.4 ± 23.5	0.466
Missed polyp,	0.4 ± 0.9	0.5 ± 0.9	0.206
Patients with missed polyps, n (%)			0.084
No	535(76.5)	209(71.3)	
Yes	164(23.5)	84(28.7)	

### Univariate analysis of risks of missed diagnoses of colorectal polyps

In the training cohort, 699 patients were divided into the missed diagnosis group (n = 535) and the non-missed diagnosis group (n = 164) based on whether they had a missed diagnosis. Univariate logistic regression analysis showed that age, sedation colonoscopy, endoscopist experience, bowel preparation, retroflected view, withdrawal time, number of polyps found on the first colonoscopy, number of polyps in the right colon, number of polyps in the left colon, number of polyps ≥ 6 mm, and number of protruding polyps were potential influencing factors for missed diagnoses of colorectal polyps (Table 2).

**Table 2:** Univariate logistic regression analysis based on the training cohort

Variable	Patients without	Patients with	*OR (95%CI)*	*P*
	missed polyps	missed polyps		
	(n = 535)	(n = 164)		
Age (years), $\overline{x} \pm SD$	52.6 ± 12.1	57.0 ± 12.8	1.03(1.01–1.05)	< 0.001
Sex, Male/Female	339/196	102/62	0.95(0.66–1.37)	0.786
Symptom, Yes/No	375/160	122/42	1.24(0.83–1.84)	0.289
Family history of CRC, Yes/No	37/498	15/149	1.36(0.72–2.54)	0.342
Diverticulum history, Yes/No	37/498	16/148	1.46(0.79–2.69)	0.232
Sedation colonoscopy, Yes/No	219/316	53/111	0.69(0.48–0.99)	0.048
Endoscopist’s experience				
500 − 1000/ ≥ 1000	120/370	50/87	1.77(1.18–2.66)	0.006
< 500/ ≥ 1000	45/370	27/87	2.55(1.50–4.34)	0.001
Bowel preparation, Poor/Adequate	85/450	49/115	2.26(1.50–3.39)	< 0.001
Retroflected view, No/Yes	472/63	156/8	2.60(1.22–5.55)	0.013
Withdrawal time, < 6/ ≥ 6	195/340	83/81	1.79(1.26–2.54)	0.001
No. of Polyps detected at first colonoscopy, ≥ 2/ < 2	260/275	112/52	2.28(1.57–3.30)	< 0.001
No. of Right colon polyps, ≥ 2/ < 2	63/472	42/122	2.58(1.66–4.00)	< 0.001
No. of Left colon polyps, ≥ 2/ < 2	85/450	38/126	1.60(1.04–2.46)	0.033
No. of Rectal polyp, ≥ 2/ < 2	32/503	17/147	1.82(0.98–3.37)	0.057
No. of Polyps < 6 mm, ≥ 2/ < 2	67/468	19/145	0.92(0.53–1.57)	0.749
No. of Polyps ≥ 6 mm, ≥ 2/ < 2	113/422	78/86	3.39(2.34–4.90)	< 0.001
No. of Flat polyps, ≥ 2/ < 2	60/475	20/144	1.10(0.64–1.89)	0.730
No. of Protruding polyps, ≥ 2/ < 2	177/358	83/81	2.07(1.45–2.96)	< 0.001
Interval	20.8 ± 23.6	24.5 ± 26.5	1.01(0.99–1.01)	0.089

### Multivariate analysis of risks of missed diagnoses of colorectal polyps

**Fig. 1:**
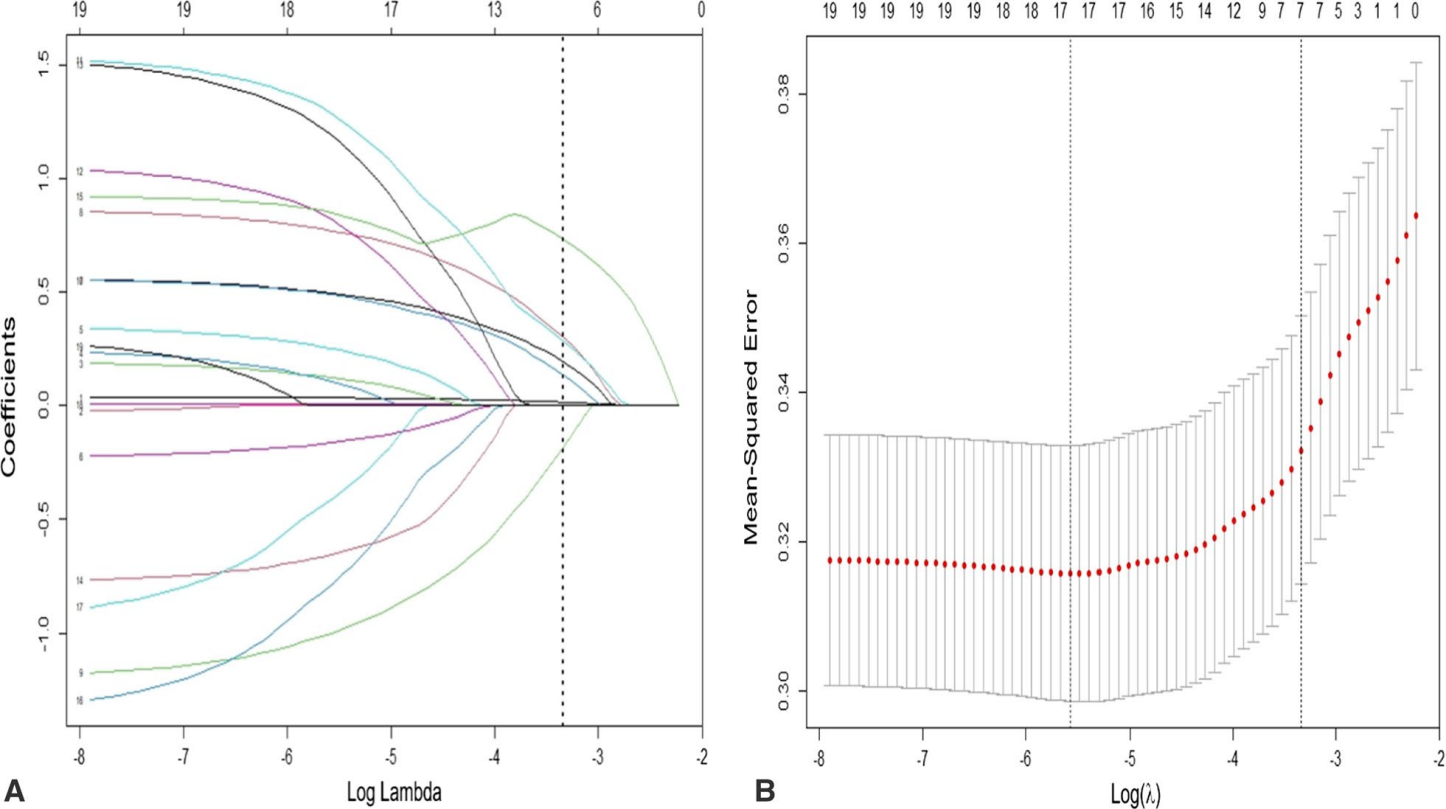
Selection of predictors using Lasso regression. **A** Lasso coefficient profiles of all clinical features. **B** Optimal penalization coefficient λ identification in Lasso model (tenfold cross validation and 1 se criterion

In the training cohort, multivariate logistic regression analysis was conducted by considering whether the patient was subject to a missed diagnosis as a dependent variable and potential influencing factors as independent variables. The results showed that age (odds ratio [OR]: 1.0; 95% confidence interval [CI]: 1.02–1.05), endoscopist experience (500–1000 cases [OR: 2.00; 95%CI: 1.27–3.13], < 500 cases [OR: 3.22; 95%CI: 1.73–6.01]), bowel preparation (OR: 2.52; 95%CI: 1.60–3.96), retroflected view (OR: 2.52; 95%CI: 1.11–5.74), withdrawal time (OR: 1.59; 95%CI: 1.07–2.36), number of polyps in the right colon (OR: 2.11; 95%CI: 1.21–3.68), and number of polyps ≥ 6 mm (OR: 2.96; 95%CI: 1.76–4.98) were independent influencing factors for missed diagnoses of colorectal polyps, as shown in Table 3. The LASSO regression results showed that the optimal parameter was λ = 0.03539431, in which case the independent influencing factors screened were consistent with the logistic regression analysis results, as shown in Fig. 1, thus proving our nomogram model.

**Table 3:**
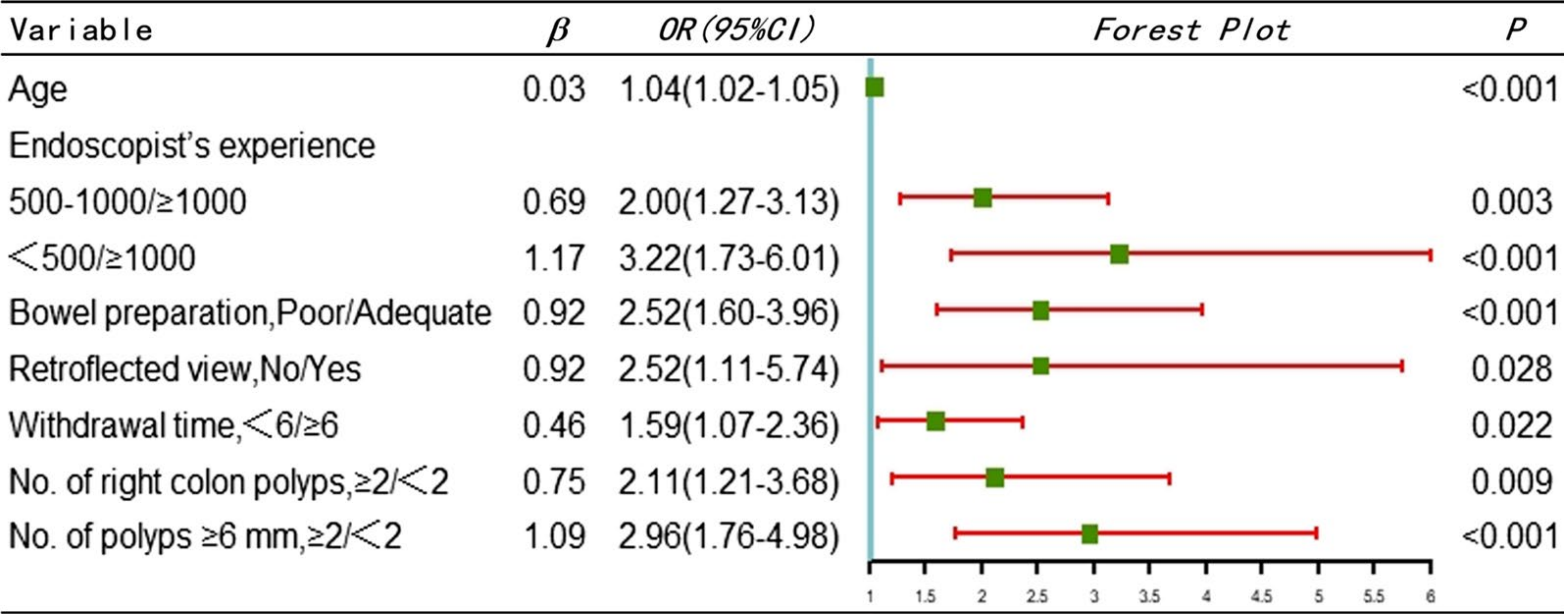
Multivariate Logistic regression analysis based on the training cohort

### Establishment of the nomogram predicting the risk of missed diagnoses of colorectal polyps

**Fig. 2:**
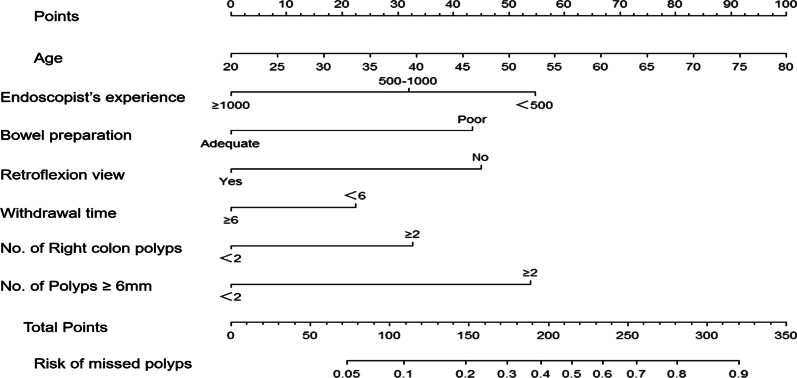
Nomogram predicting the risk of missed diagnoses of colorectal polyps

According to the multivariate logistic regression analysis, seven independent risk factors were used to construct a nomogram predicting the risk of missed diagnoses of colorectal polyps (Fig. 2). The score of each independent influencing factor was the point of the corresponding scoring scale, and the total points for each subject were the sum of the scores of each independent influencing factor. The value corresponding to the total points on the colorectal polyp missed diagnosis risk axis corresponded to the risk of a missed diagnosis of colorectal polyps. The higher the number of points, the higher the risk of missed diagnoses of colorectal polyps.

### Validation of the nomogram predicting the risk of missed diagnoses of colorectal polyps

**Fig. 3:**
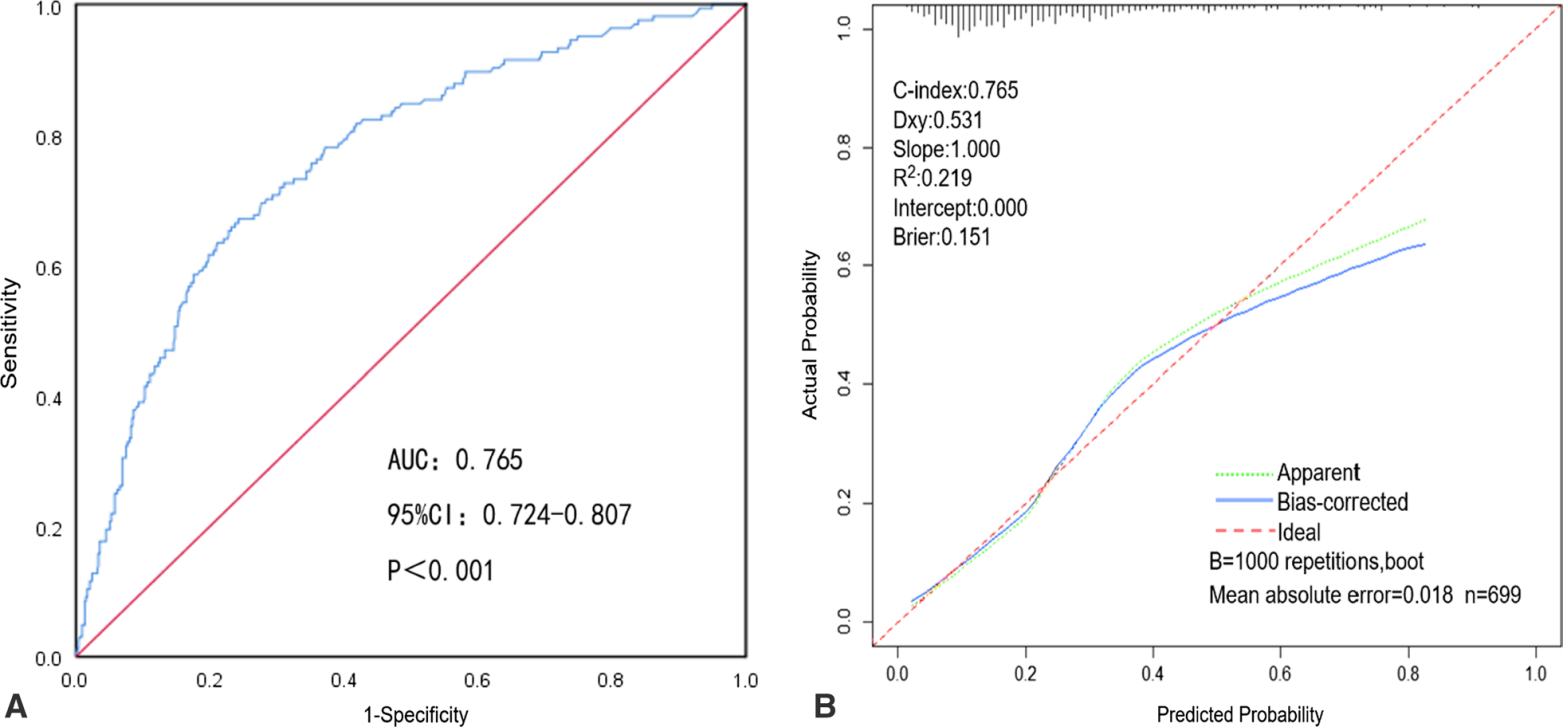
Analysis of discrimination and calibration of the nomogram in the training cohort. **A** Receiver operating characteristic curve of the nomogram in the training cohort. **B** Calibration curve of the nomogram in the training cohort

**Fig. 4:**
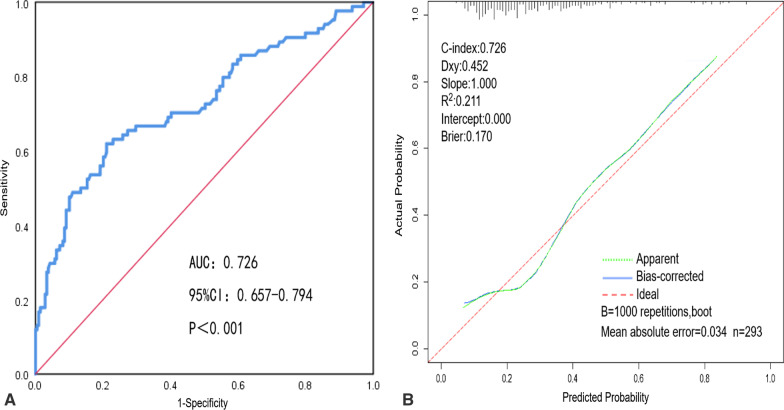
Analysis of discrimination and calibration of the nomogram in the validation cohort. **A** Receiver operating characteristic curve of the nomogram in the validation cohort. **B** Calibration curve of the nomogram in the training cohort

In the training cohort, the nomogram showed good discrimination and calibration in predicting the risk of missed diagnoses of colorectal polyps, with both the C-index and AUC of 0.765 (95%CI: 0.724–0.807). This suggests that the nomogram had a good discrimination ability (Fig. 3A). The calibration curve showed good consistency between the risk of missed diagnoses of colorectal polyps predicted by the nomogram and the actual risk of missed diagnoses obtained by two colonoscopies (Fig. 3B). In the validation cohort, the nomogram also showed good discrimination and calibration in predicting the risk of missed diagnoses of colorectal polyps, with both the C-index and AUC of 0.726 (95%CI: 0.657–0.794) (Fig. 4A). There was also a good calibration curve between the predicted and actual risk of missed diagnoses (Fig. 4B).

### Analysis of the clinical efficacy of the nomogram in predicting the risk of missed diagnoses of colorectal polyps

The optimal cut-off value for the total points of the nomogram calculated using the Youden index was 152.2 points, and patients with a total score ≥ 152.2 points were classified as high-risk, while patients with a total score < 152.2 points were classified as low-risk. Under the cut-off value, the sensitivity, specificity, positive predictive value, negative predictive value, positive likelihood ratio, and negative likelihood ratio were 67.1%, 75.7%, 45.8%, 88.2%, 2.8, and 0.43, respectively, in the training cohort, and 57.1%, 79.9%, 53.3%, 82.3%, 2.8, and 0.54, respectively, in the validation cohort (Table 4).

**Table 4:** Analysis of clinical efficacy of the nomogram

Variable	Training cohort (95%CI)	Validation cohort (95%CI)
C-index	0.765 (0.724–0.807)	0.726 (0.657–0.794)
Cut-off score	152.2	152.2
Sensitivity, %	67.1 (59.3–74.2)	57.1 (45.9–67.7)
Specificity, %	75.7 (71.8–79.3)	79.9 (73.7–85.0)
Positive predictive value, %	45.8 (40.9–54.4)	53.3 (42.6–63.8)
Negative predictive value, %	88.2 (84.3–90.2)	82.3 (76.2–87.1)
Positive likelihood ratio	2.8 (2.3–3.3)	2.8 (2.0–3.9)
Negative likelihood ratio	0.43 (0.35–0.54)	0.54 (0.42–0.69)

### Clinical decision curve analysis of the nomogram predicting the risk of missed diagnoses of colorectal polyps

**Fig. 5:**
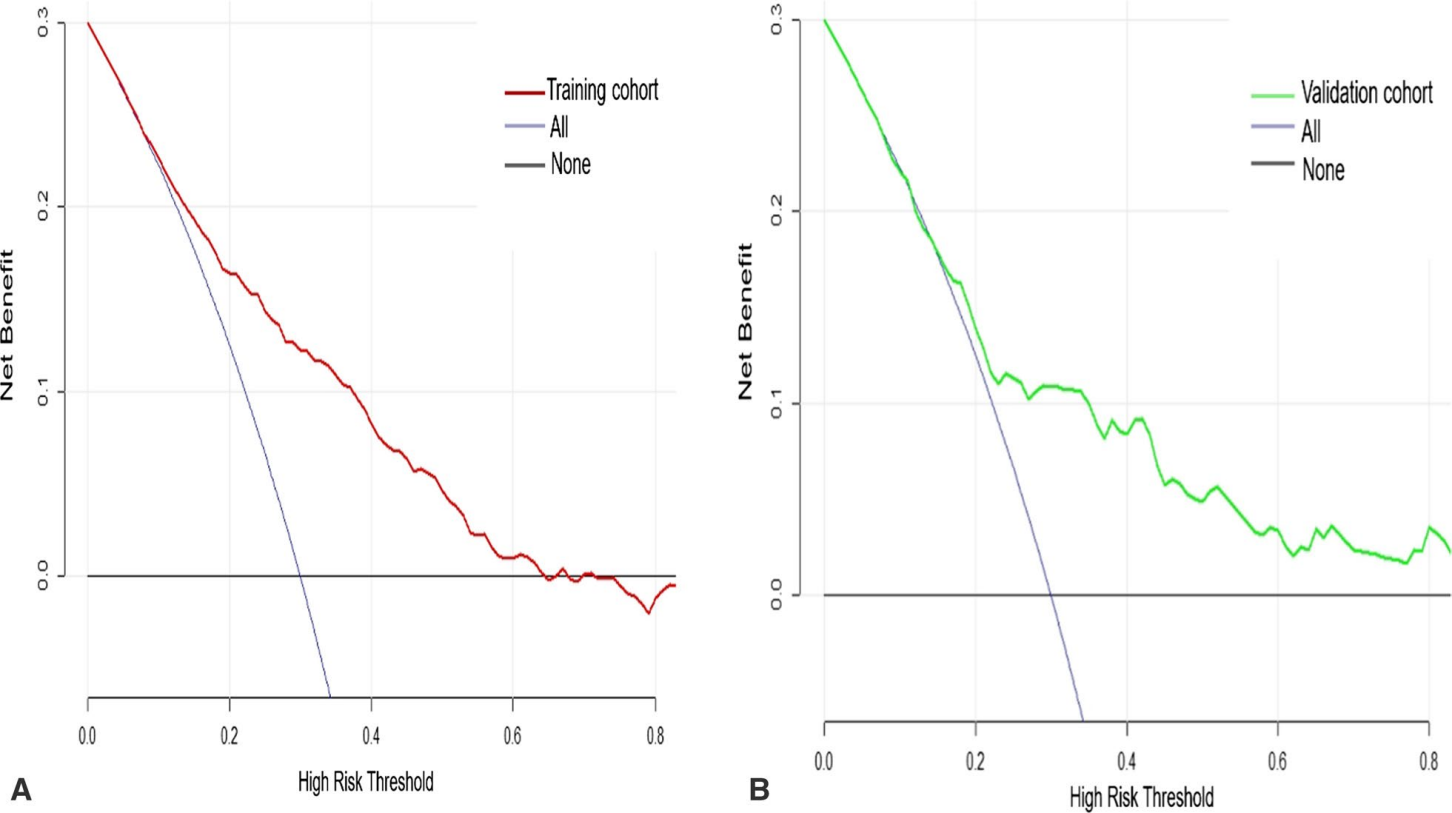
Clinical decision curve analysis of the nomogram. Y-axis represents net income; X-axis represents the threshold probability; the red line represents the net income of the nomogram model in the training cohort; the green line represents the net income of the nomogram model in the validation cohort; the black line indicates no second colonoscopy for all patients, and the grey line assumes a second colonoscopy for all patients. **A** Clinical decision curve analysis of the nomogram in the training cohort. **B** Clinical decision curve analysis of the nomogram in the validation cohort

The clinical decision curve analysis was used to analyse the net income of the nomogram under different threshold probabilities to evaluate the clinical application value of the nomogram. As shown in Fig. 5, the training cohort was within the threshold probability range of 0.15–0.65, while the validation cohort was within the threshold probability range of 0.17–0.80. Using this model to identify patients with missed diagnoses had the edge over the scheme of ‘no second colonoscopy for all patients’ or ‘second colonoscopy for all patients’. In other words, using this model to predict missed diagnoses of polyps could benefit some patients.

## Discussion

Polyps or adenomas missed in colonoscopy may lead to the development of interval colorectal cancer [12, 13]. However, there is a lack of studies to predict the risk of missed colorectal polyps in each individual patient. In this study, univariate and multivariate logistic regression analysis indicated that age, endoscopist experience, bowel preparation, retroflected view, withdrawal time, number of polyps in the right colon, and number of polyps ≥ 6 mm were independent factors for missed diagnosis of polyps among patients. We developed and validated a nomogram that predicts the risk of missed colon polyps after colonoscopy using these factors.

Shin et al. [8] reported that the risk of missed diagnoses of adenomas in patients aged ≥ 60 years was twice that in patients aged < 60 years. It may be because, with ageing, the curvature and folds of the colon gradually increase as well as the frequency of colonic diverticula [14], making polyps harder to detect. Our results are consistent with this. In this study, the risk of missed diagnoses of polyps in patients with poor bowel preparation was 2.52 times that in those with good bowel preparation. Multiple previous studies have shown that high-quality bowel cleansing can help significantly reduce the missed diagnosis of polyps [15, 16]. A study [17] on 104,618 colonoscopies conducted by 201 endoscopists showed that the detection rate of adenomas varied greatly between endoscopists, ranging from 6.3% to 58.7%. Yao et al. [18] also showed a significant correlation between the experience of endoscopists and missed diagnoses of adenomas. Our study also found that the more experienced the endoscopist, the lower the risk of missed polyps. The withdrawal time of the colonoscope is an important indicator of the quality of the colonoscopy. Sufficient withdrawal time guarantees careful observation of the colonic mucosa. Several previous studies have confirmed that the longer the withdrawal time, the higher the detection rate of adenomas [19–21]. In our study, patients with a withdrawal time of fewer than six minutes had 1.59 times higher risk of missed polyps than those with a withdrawal time of ≥ 6 min.

Compared with the left colon, the rate of missed diagnoses of adenomas in the right colon was significantly higher, and the rate of missed diagnoses of adenomas in the caecum and ascending colon was 35.6% [22]. Several studies [23–25] have reported that multiple adenomas, flat adenomas, and sessile serrated adenomas/polyps are mainly located in the right colon, are small in size, are covered with mucus, and are not easily detected during colonoscopy. In addition, the folds of the right colon are deep and difficult to unfold, which results in poor visualization during endoscopy, which may also be one of the reasons why right colon adenomas are easily missed. Similar results were obtained in our study. Previous studies [26, 27] found that using a retroflected view in colonoscopy improved the detection of adenomas, especially in the right colon. Pickhardt et al. [28], in a study on the location of missed adenomas, 14 of 15 non-rectal missed adenomas (93.3%) were located on the folds, and 10 (71.4%) were located near the plica opening. However, in conventional colonoscopy, it is difficult to detect adenomas in these areas by front-view observation, while a retroflected view can help effectively observe these blind areas in the field of vision, improve the detection rate of adenomas, and reduce the rate of missed diagnoses [29]. This is consistent with our research results. Interestingly, our study showed that patients with ≥ 2 polyps larger than 6 mm in diameter were 2.96 times more likely to miss polyps than those with less than two polyps larger than 6 mm in diameter. A study found that patients with small adenomas (6–9 mm) are more likely to develop multiple adenomas than those with small adenomas (< 5 mm) [11]. Kim et al. [7] also found that patients with multiple adenomas had a higher risk of missed diagnoses of adenomas. A possible reason was that after the endoscopist detected a certain number of adenomas, they were not as focused as before.

The advantages of this study are as follows. First, previous studies [6, 8] mainly focused on a single polyp as the research object to analyze the risk factors of missed polyp diagnosis. However, these studies failed to distinguish which patients had missed polyps after colonoscopy. This study takes a single patient as the research object, analyzes the risk factors of polyp missed diagnosis after colonoscopy and constructs a model to evaluate the risk of missed diagnosis of a single patient, which may make up for the shortcomings of previous studies. Second, after completion of colonoscopy, the nomogram established in this study could quickly assess the risk of missed diagnoses of colorectal polyps in the patient and provide possible risk factors, and endoscopists could formulate appropriate and timely follow-up strategies for patients based on the prediction results. Third, the nomograms were internally validated using bootstrap methods and externally validated using validation datasets, and both show good discrimination ability.

The limitations of this study are as follows. Firstly, this is a retrospective study with possible selection bias, and the sample size of this study is small, so a larger, multicenter prospective study is needed to verify the model's validity. Secondly, some new types of endoscopes that can improve the detection rate of adenomas, such as high-definition colonoscopes, wide-angle colonoscopes, panoramic endoscopes, and virtual staining endoscopes, were not used in this study, which might have affected the results of this study. Finally, we failed to assess the risk factors for missed colorectal polyps in patients with different pathological types.

## Conclusions

The study showed that age, the endoscopist's experience, bowel preparation, retroflected view, withdrawal time, number of polyps in the right colon, and number of polyps ≥ 6 mm were independent influencing factors for missed diagnoses of colorectal polyps. The nomogram established based on these independent influencing factors showed good discrimination, calibration, and clinical efficacy. It provides a reference value for endoscopists and physicians to intuitively and conveniently analyse the risk of missed diagnoses of colorectal polyps in patients, identify high-risk groups, and formulate appropriate and timely follow-up strategies.

### Clinical practice points

This study was based on clinical data from 992 patients to analyse the risk of missed diagnosis in individuals during colonoscopy, and independent risk factors for missed diagnoses included age, endoscopist’s experience, bowel preparation, retroflected view, withdrawal time, number of polyps in the right colon, and number of polyps ≥ 6 mm, were identified. These seven independent risk factors were used for the first time to construct a nomogram for predicting the risk of missed diagnoses. The C-index of the nomogram in the training and validation cohorts was 0.763 (95%CI: 0.724–0.807) and 0.726 (95%CI: 0.657–0.794). The nomogram provides a reference value for clinicians to analyse the risk of a missed diagnosis of colorectal polyps in individuals, identify high-risk groups, and formulate appropriate follow-up strategies.

## Data Availability

The datasets used and analysed during the current study are available from the corresponding author on reasonable request.

## Abbreviations

AUC: Area under the receiver operating characteristic curve
CI: Confidence interval
OR: Odds ratio
